# Protecting the normal in order to better kill the cancer

**DOI:** 10.1002/cam4.488

**Published:** 2015-07-14

**Authors:** Bingya Liu, Lewis Ezeogu, Lucas Zellmer, Baofa Yu, Ningzhi Xu, Dezhong Joshua Liao

**Affiliations:** 1 Shanghai Key Laboratory of Gastric Neoplasms, Ruijin Hospital, Shanghai Jiao Tong University School of MedicineShanghai, 200025, China; 2 Hormel Institute, University of MinnesotaAustin, Minnesota, 55912; 3 Beijing Baofa Cancer Hospital, Shahe Wangzhuang Gong Ye YuanChang Pin Qu, Beijing, 102206, China; 4 Laboratory of Cell and Molecular Biology, Cancer Institute, Chinese Academy of Medical ScienceBeijing, 100021, China

**Keywords:** Cancer, Chemotherapy, Chemoresistance, Mutation, Metastasis

## Abstract

Chemotherapy is the only option for oncologists when a cancer has widely spread to different body sites. However, almost all currently available chemotherapeutic drugs will eventually encounter resistance after their initial positive effect, mainly because cancer cells develop genetic alterations, collectively coined herein as mutations, to adapt to the therapy. Some patients may still respond to a second chemo drug, but few cases respond to a third one. Since it takes time for cancer cells to develop new mutations and then select those life-sustaining ones via clonal expansion, “run against time for mutations to emerge” should be a crucial principle for treatment of those currently incurable cancers. Since cancer cells constantly change to adapt to the therapy whereas normal cells are stable, it may be a better strategy to shift our focus from killing cancer cells per se to protecting normal cells from chemotherapeutic toxicity. This new strategy requires the development of new drugs that are nongenotoxic and can quickly, in just hours or days, kill cancer cells without leaving the still-alive cells with time to develop mutations, and that should have their toxicities confined to only one or few organs, so that specific protections can be developed and applied.

## Introduction

Cancer prognosis has been greatly improved these days, compared to 1971 when the US president Nixon declared the war on cancer 1. Some cancer types are basically curable, such as testicular cancer, gestational choriocarcinoma, and some subtypes of leukemia. Certain cancers, including lung, colorectal, breast, and prostate cancers, have a greatly diverse prognosis, with some early-diagnosed cases curable simply by surgical removal while many other cases, especially those that miss an early diagnosis, having a dolorous outcome. Skin melanoma and nasopharyngeal cancer may also belong to this group as they may be killed by high dose of interleukin-2 2–4 and by radiotherapy with or without chemotherapy 5–7, respectively. There are some other types of cancer that may not be curable but are indolent and may take over a decade to progress to the terminal stage, such as thyroid cancer 8–10. Unfortunately, there are still certain cancers, such as pancreatic, liver, or gastric cancer, that basically have no cure and are difficult to diagnose earlier; even those cases that are diagnosed at an early stage by serendipity are very likely to die from metastases eventually. A huge number of chemotherapeutic drugs (“chemo drugs” hereafter for brevity) have been developed in the past decades. However, although some of them have magical efficacy initially, especially those having specific targets and so-called magic bullets 1,11,12, basically all of them will eventually encounter resistance 13 after having given an initial high to the patient and sometimes also to the oncologist. Even worse, these drugs will induce resistance via not only quick, nongenetic mechanisms 14–16 but also genetic mutations and ensuing clonal selections of the cells that basically resist all kinds of remedies 17,18. After a long cogitation, we have come up with a new strategy for those currently incurable cancers and present it in this essay for peers to debate.

## Some Lethal Tumors were Curable Even Hundred Years Ago

According to Nauts' description of a book by Tanchou in 1844, Dussosoy in France inoculated an ulcerated breast cancer with gangrenous matter through a small incision or dressed the ulcers with gauze wraps soaked with gangrenous discharge, which caused high fevers and remission of the tumor 19. During 1866–1867, Busch in Germany used cotton-wool bandages to transmit bacteria from erysipelas patients onto small burn injuries of bone sarcoma patients, which caused fever and tumor remission, although complete remission without regrowth required iterations of the procedure 20–22. In 1882 Fehleisen confirmed Busch's therapy and identified *Streptococcus pyogenes* as the erysipelas-causing bacteria 23. In 1887, Bruns cured a recurrent melanoma with erysipelas and summarized 14 reported cases with complete or stable remission 24. During 1891–1936, Coley at New York injected, first live and then heat-killed, bacterial mixtures of *S pyogenes* and *Serratia marcescens* to patients with different sarcomas or certain epithelial cancers 25. Ironically, this so-called “Coley's vaccine” or “Coley's toxin” appears to have been better than any regimen used today, since about 500 of the 1000 patients so treated by Coley or others showed tumor regression with many, including 2 of 4 ovarian cancer patients, surviving over 20 years 26,27. Its last use in the late 1980s, which happened to be in China, resulted in a complete regression of a terminal liver cancer 28,29. Likely, these bacterial remedies are not only immunotherapies but also work through hyperthermia, as the efficacy largely depends on whether the patients responded with a high fever 25 and as hyperthermia acts largely by stimulating immune function as well 30. Unfortunately, these ancient remedies are no longer used, in our opinion because (1) fever tortures the patients with great discomfort, (2) it is easier to standardize chemo drugs and radiation instead, (3) today those bacterial strains may be less virulent while the population's immune response may be different, compared to over a century ago, due to wide vaccine and antibiotic uses, (4) different preparations of the bacteria had different efficacies, and (5) bacteria as natural substances are not patentable and thus unattractive to the drug industry 27,31. Moreover, these ancient remedies may be unethical today in many countries.

Actually, Deidier had noticed in 1725 that tumors of syphilitic patients were cured more often than others and that prostitutes infected with syphilis had a lower frequency of cancer than the average population 22. Trnka de Krzovitz also reported in 1783 that development of tertian malaria could cause complete regression of breast cancer in a few weeks 29,32. In 1899, D'Arcy Power already observed an inverse correlation of malaria to cancer, and wrote “where malaria is common, cancer is rare” 33. These historical reports on the inverse correlation of acute febrile infections to cancers, as reviewed by Hoption Cann 25,34, show that spontaneous cure of cancer was much more commonly seen hundred years ago than today. Since 1860s when aseptic technology for surgical dressings and steam sterilization of surgical implements emerged, surgery-caused acute febrile infection has become uncommon. In 1899, aspirin emerged, followed by many other antipyretics. The dramatic diminution of acute febrile infection occurred after 1940s when many antibiotics started to emerge. These medical advances are thought to account significantly for the continuous drop of the frequency of spontaneous cure of cancers 35–39. Supporting this conjecture, acute febrile infection has been associated with many cases of spontaneous cancer regression 22,33–36,39–47 and prophylaxis 37,38,48–52, as reviewed recently 18.

The above described ancient cases, even if some of them might have been misdiagnosed, undeniably prove that some cases of malignancies, even those that today are still highly lethal, such as bone sarcomas, were once curable. Moreover, these ancient cases also show that historically, drug discovery was made through serendipitous clinical findings followed by search and evaluation of scientific literature. Even chemo drug development was commenced in this way 18,53. However, roughly since 1929 when penicillin was discovered and especially since the 1950s when more antibiotics were discovered and some chemo drugs were developed 54,55, drug development has been shifted to an “R&D” (research and development) mode, that is, from laboratories to clinics. Today, this mode “is presently experiencing an insufficiency crisis” as put by Kienle 31, or in our opinion is very wasteful in resources, efforts and time.

## New Chemo Drugs Should be Nongenotoxic and Should Make it Harder for Cancer Cells to Develop Resistance

Modern chemo drug development has experienced three waves 56. The first wave was centralized on targeting fast-proliferating cells because it has been, until now, a widespread misconception that cancer cells are fast-proliferating. Actually, most cancer cells, especially those in highly lethal solid tumors, proliferate relatively slowly, but a tumor mass often has a large portion of cells at the proliferating status and thus manifests a fast enlargement, as explained recently 57,58. In contrast, many normal cells in such tissues as bone marrow, hair-follicles, gastrointestinal (GI) tract, and epidermal skin proliferate much faster than most cancer cells 57,58. For this reason, chemo drugs developed in the first wave have severe side effects on these normal fast-proliferating cells 18,53, exhibited as cytopenia, alopecia, skin itch, nausea, vomiting, and diarrhea, etc., although some drugs 59 such as 5-fluorouracil (5FU) 60 may stimulate certain immune cells that have anticancer activities. Today, many of these classic chemo drugs, including 5FU and cisplatin as well as their newer versions, are still the major ones used clinically. The second wave is so-called “targeted therapy” that targets a specific component of a growth- or survival-pathway. A new version of targeted therapy is based on a so-called “synthetic lethality” principle 61. As an example, cancer cells may have lost a DNA-damage-repair mechanism and thus are completely dependent on an alternative pathway 62. Compromising this alternative will induce cancer-cell-specific death while sparing normal cells in which this pathway is functionally redundant 63. Most chemo drugs developed in these two waves are mainly genotoxic, not only because they mainly target genomic DNA or some components that affect or are needed for DNA synthesis or repair 64 but also because most mutations are deleterious 65. The same severity of genotoxicity causes more severe DNA damage in cancer cells than in normal cells, thus eliciting cancer cell-specific killing, because normal cells have intact DNA damage response and intact DNA repair mechanism and thus can arrest proliferation for more efficient DNA repair and to avoid damage by the drug. However, the impairment in these two mechanisms also allows cancer cells to quickly accumulate mutations and, with these mutations, cancer cells can easily establish a bypass to circumvent the target, thus developing therapy resistance 18. Generally speaking, most clinically used chemo drugs provide only 9–14 months of effect before resistance occurs 13,58,66. The third wave of drug development targets some cytoplasmic components the functions of which not only are changed in cancer but also cannot be carried out by other cellular effectors, thus giving cancer cells more difficulties in developing resistance 56. The targets of this new wave may be normal gene products that are aberrantly expressed 67, unlike many in the second wave that are mainly mutants. Controls of osmotic pressure, oxygen partial pressure, cellular acidity, and ion channels are among these targets 68–73. Heat shock proteins that protect cancer cells not only from hyperthermia but also from many other forms of stress 74,75 are the targets as well, because usually the levels of heat shock proteins are already high in cancer cells before heat stress or various therapies and thus may not be further raised during a therapy to protect the cancer cells 56,72,76,77.

## Targeting Nongenomic Components While Protecting Normal Cells may be a Better Chemotherapeutic Strategy

In a sharp contrast to normal cells that firmly maintain their genomic integrity, cancer cells constantly mutate their genomic DNA to adapt to the environment, especially during and after a therapy. While they are killing cancer cells, radio- or chemo therapies also damage the DNA of the remaining cells and impose onto the cells a pressure to select out the resistant clones 78,79. To better resist a hostile environment, some cancer cells not only withdraw from a cycling status to a dormant (i.e., G0) one but also manifest a stem-cell-like expression profile by turning off the expression of most, if not all, cell-specific markers. Because of these features, these stem-cell-like cancer cells, which were often observed in the areas called “stem cell nitches,” are refractory to currently available treatments. Therefore, refocusing onto how to protect the more-stable normal cells from the toxicity of chemotherapy, instead of on how to kill the always-changing cancer cells per se, may be a better strategy to cure cancer, as having been proposed by Blagosklonny and Pardee previously 80, although the purpose still would be to hit cancer cells more harshly. In our rumination, this new strategy requires development of new chemo drugs or therapeutic implementations that act mainly via nongenomic mechanisms and can kill cancer cells very quickly, in just hours or days in patients, unlike most currently used drugs that are genotoxic and require weeks or months of treatment 18. This is doable, as some chemo drugs can kill cancer cells in just thirty minutes in culture dishes 81 and as hyperthermia can kill cancer cells in just hours or days in patients 41. Of the above described ancient cases, development of tertian malaria could cause complete regression of breast cancer in a few weeks 29,32 and the breast cancer ulcers treated by Dussosoy with gangrene became bright red in a few days and the tumor sloughed off at the 19th day post the first treatment 19. The sarcomas treated by Busch with bacteria regressed within 2 weeks 20–22. Coley's vaccine could cause an evident effect on some sarcomas the next day after the first injection and could cause complete remission of the tumors in 2 weeks 19,27,31. Actually, the tumors should become pale and softened within the first one to several days of the treatment; otherwise the efficacy of the vaccine would be poor 27,31.

A second requirement of our new strategy is that the effects of the new drugs or implementations should be transient. Of the common detrimental effects of chemotherapy, nausea, vomiting, and diarrhea usually cease soon after the treatment is terminated, suggesting that the GI tract mucosa can recover very quickly. However, hair regrowth occurs about 1–6 months, in some cases much later, after termination of the chemotherapy 82, which reflects a visible long-lasting effect of the chemo drugs. Myelosuppression, especially the neutropenia, often could last for many years and in many cases could even be permanent (irreversible) 83–85. Likely, many chemo drugs exert long-lasting calamities on some other normal cell types as well, although systematic studies on them are still lacking. An interesting question is thus raised as to whether the therapeutic effects of some chemo drugs also persist long after the therapy has been terminated. Peculiarly, this important question has hardly been addressed hitherto although there are inklings of such persistence in clinical case reports. We surmise that some effects of some chemo drugs on some cancers also last long after cessation of the therapy but likely are not perpetual, in part because cancer cells may later develop resistance. The persistency on one hand may allow a decrease in dose or treatment duration, which is good for the patient, but on the other hand may also become an abiding propulsion for the evolution of the remaining cancer cells to more-untoward statuses, including therapy resistance and metastasis. It is our conjecture that nongenotoxic drugs may lack, or have a shorter period of, long-lasting effects on both normal and cancer cells. It is better for our therapeutic strategy to use those drugs without such persistency to avoid prodding the remaining cancer cells to more-menacing phenotypes.

A third crucial requirement of our strategy is that the injurious effects of the new drugs or therapeutic implementations need to be confined to one or to just a few organs or tissues, so that specific protection can be developed and applied, such as by a protective drug or by placing the patient in an intensive care unit (ICU) or an operating room with all its sophisticated devices. For example, cardiopulmonary bypass can be used to protect the heart and lung, or other bypass approach to protect other organs, for a short period of time. Clinical trials on camptothecin sodium for cancers in the 1970s were dropped due to its severe nephrotoxicity 86,87, and a main calamity of cisplatin is also nephrotoxicity 88. Preventing such organ-specific toxicity should be feasible, theoretically, although development of protective drugs or approaches for broader mishaps may be difficult. Corcos has proposed a “cell inflation” hypothesis in which increase (“inflation”) in the number of normal cells is expected to decrease, via a negative feedback loop, the proliferation of normal cells, and in turn decrease the toxicity of a chemo drug. In his interesting hypothesis, increase in the number of granulocytes by such as transfusion may be a universal cancer therapy, given the fact that myelosuppression is a common adversity of chemotherapy 89. Conversely, Blagosklonny and Pardee have suggested that induction of the p53- and p21-expression by a low dose of the genotoxic chemo drug doxorubicin should be able to arrest and in turn protect normal cells as well 80. Some drugs such as UCN-01 that more easily arrest growth of normal epithelial cells, thus seemingly unsuitable to be chemo drugs, may instead be used to inhibit angiogenesis and thus inhibit cancer growth 80,90.

## The First Blow on a Cancer Should be Hard, but the Later Ones May be Soft

One of the things we have learned from the aforementioned ancient cases treated by Dussosoy, Busch, Coley, and others with severe acute bacterial infections is that we should strike brutally at cancers without mercy, that is, punch them with the hardest blows or with the maximal tolerated doses (TMD) in today's term. Since it takes time for cancer cells to develop mutations and then select out the survival-sustaining ones via clonal expansion, deprival of such time from cancer cells becomes crucial. The hardest punch with the upcoming new nongenotoxic drug aims to kill as many cancer cells as possible in the shortest time period, albeit the patients are likely to be hit heavily as well, as seen in those ancient patients. Therefore, one crucial principle of our strategy is to “run against time” to leave the remaining cancer cells with no time (1) to withdraw from a cycling status to the G0 phase (i.e., a dormant status) and (2) to develop mutations and then select out the resistant mutants 18. To make the treatment short, the TMD of the upcoming drugs should probably be much higher than the routinely defined one and may risk the patient's life, which contrasts with the mainstay of today's chemotherapy that tends to be nontoxic to the patients. Moreover, protection of the normal organ(s) in an ICU, if it is necessary, and management of some possible oncological emergencies such as acute tumor lysis syndrome 91, are applicable only for a short time period. The nongenotoxic nature of the new drugs or remedies and the short duration of the treatments are expected to have little persistency of side effects, especially the myelosuppression.

While the new drugs or implementations developed according to the criteria described above may be curative for many cancers when given at TMD, certainly in a number of cases some cancer cells will survive the initial tough treatment and eventually develop to recurrent or metastatic tumors, likely after a latency that is longer than usual. In this situation, the TMD may not be used again, no matter with the same drug or another one. Instead, an adaptive therapy, that is, a low dose of chemo drug 78,92, should be used to avoid or delay not only therapy-induced resistance but also therapy-driven invasion and metastasis, because a harsher treatment is also a stronger impetus for progression via mutations and selections 93,94. According to what we have learned from cancer ecology 95, an environment with a low drug concentration is slightly hostile but may still be acceptable to the cancer cells. In such an environment, cancer cells may shift from a fast-proliferating status to a slowly proliferating or even dormant one, because cells that grow more slowly survive better 93, but the cells may not have motivations to disperse, that is, to invade locally or metastasize distantly. This may make a low dose of chemo drug effective via a so-called “Darwinian bypass” principle 96. Of course, the dose that can create such a “slightly hostile but still acceptable” environment varies among patients and is difficult to establish, although it is required.

Calorie restriction 97–100 and even insulin potentiation therapy 101,102 may be considered as an adjuvant to our new approach, not only to decrease the toxicity to the normal cells but also to potentiate the efficacy on the cancer cells and to prevent progression toward more-dreadful forms. This is because cancer cells consume a much larger amount of glucose than normal cells 103 and produce a highly acidic microenvironment that in turn makes cancer cells more malignant 104,105. The now-discredited report 106 of converting blood cells from adult mice to pluripotent stem status simply by culturing the cells in an acidic medium, while apparently invalid in its research, does add discussion to a concept that makes sense in tumor biology, since on many occasions a phenotype of more malignancy reflects a cell type more similar to stem cells.

## Pairing Normal with Cancerous Cell Lines in Chemo Drug Studies Means Little and May Let Some Promising Candidates Slip Away

As aforementioned, drug development has been shifted to an R&D mode, which consists of three steps, that is, (1) in vitro study with cell lines in culture dishes, (2) in vivo study with animals, in most cases using xenograft models wherein human cancer cells are inoculated into immunodeficient mice to allow tumor development, and (3) clinical trials in humans. In the first step, that is, in the in vitro studies, cancer cells are often paired with “normal” cell lines from the same tissue or organ of the cancer, although the immortalized cell lines are not really normal and often have features of benign tumors 107. If the drug tested is less toxic to the normal than to the cancer cell lines, it is explained to have a potential therapeutic window clinically. This explanation seems to make sense at the first hearing, but it actually has little clinical relevance and is a misconception, because clinically toxicity of a chemo drug is mainly concerned on three cell types that in most cases are not those from which cancers are developed: The first type is those fast-proliferating normal cells as described in a previous section. The common side effects of chemotherapy such as low blood cell counts are ascribed to the toxicity to these cells. The second type is those cells that have undergone terminal differentiation and have lost their regeneration capacity, with neurons and heart muscle cells as good examples 91,108. Toxicity to these cells, for example, neuro- and cardiac toxicity, has serious consequences because they are no longer able to regenerate. The third type is those important metabolic organs, typically the liver and kidneys, that metabolize the drug and thus often are hit severely by it. In contrast, usually the parental cell type from which a cancer is developed is not a practical concern during chemotherapy. For instance, whether a chemo drug also equally kills mammary or prostate epithelial cells during the chemotherapy of breast or prostate cancer is not a concern. Actually, even if the killing by an agent is not cancer-cell specific, the agent may still be used as a so-called “therapeutic warhead” by being linked to an antibody or a protein that is specifically expressed in, and thus can guide the agent to, cancer cells 109–111. Moreover, our new strategy described above allows severe but target-confined toxicity. For these two reasons, this widespread misconception may mistakenly let some promising drugs slip away.

Another pitfall is that normal epithelial cell lines retain some features of differentiation and their optimal growth in dishes requires some special substances. For example, glucocorticoid hormones are required for the growth of mammary epithelial cell lines 112,113. However, in many drug studies, “normal” epithelial cell lines are cultured using the same medium as for the cancer cell lines without considering the special needs for the normal lines. Inappropriate culture of the “normal” cells may make it easier or harder for them to be killed by the tested agent than the cancer cells, leading to biased conclusions. Conversely, since some supplementary substances can enhance or inhibit the death of cancer cells via a stress-induced-cell-death (SICD) mechanism, which is commonly mistaken as apoptosis 18, it is also inappropriate to culture cancer cells with a medium conditioned for the normal cells. For instance, the potency of glucocorticoids in inhibition of SICD of normal and cancerous breast epithelial cells may be different 114–117, especially when the BRCA1 gene is mutated 118. Cancer cells have many mutations and thus may differ from their normal counterparts in the response to different supplementary substances in the culture medium.

## Conclusions

Some cancers have a good prognosis or a high chance of being cured while some others have a dolorous outcome, even when they are diagnosed at a relatively early stage. Therefore, when facing a cancer patient, an oncologist needs to decide whether the tumor is one of the former or one of the latter. If it is a case of the former, an already available remedy should unquestionably be given. If it is a case of the latter, an available remedy may prolong the survival but in the meantime may goad some cancer cells to develop mutations, some of which constitute the genetic bases for the upcoming contumacious phenotypes. In this regard, the so-called “disease-free” or “overall” survival, even if it is a relatively long one, is obtained at an expense of the patient's future life, and thus should be explained to the patient. Since cells of those highly lethal cancers are always mutating their genes to adapt to a therapy, whereas normal cells always try hard to maintain their genomic integrity, it may be a good idea to shift our focus from the traditional thinking of how to kill cancer cells per se to how to protect normal cells from the toxicity of a particular chemotherapy. This new strategy requires development of new nongenotoxic chemo drugs or new remedies that can quickly, in just hours or days, kill cancer cells before they develop mutations and ensuring resistance. Moreover, the toxicity of the new drugs or remedies should be confined to only one or few organs or tissues so that specific prevention or protection can be developed and applied. With these new drugs or remedies, cancers can be toughly treated, probably in ICUs or operating rooms where the severe toxicity can be better monitored and managed. This new concept of “strike hard at cancer in the ICU or operating room” opposes the mainstay of today's chemotherapy that tends to minimize the toxicity to the patients. If some cancer cells survive the initial unsparing treatment and have become advanced, a microdose of some chemo drugs may still be able to retain the cancer cells in a slowly proliferating or dormant status with a reluctance to further disperse, thus allowing the patients to survive for a long time.
